# Peri‐operative management and analgesic strategy for a patient undergoing quadruple limb amputation

**DOI:** 10.1002/anr3.12296

**Published:** 2024-05-01

**Authors:** L. Fenton‐May, M. Irvine, D. Huckle, P. Carter

**Affiliations:** ^1^ Department of Anaesthesia Cardiff & Vale University Health Board Cardiff UK; ^2^ Department of Anaesthesia Cardiff & Vale University Health Board Cardiff UK

**Keywords:** amputation, analgesia, regional anaesthesia

## Abstract

Inadequately managed amputation pain can contribute to postoperative morbidity and mortality. However, amputation pain can be challenging to manage due to its complex nature, with both central and peripheral nociceptive and neuropathic elements. Here, we present the case of a 47‐year old man who developed irreversible ischaemic injuries to all four limbs following admission to intensive care with sepsis. He required quadruple amputation and we describe our approach to his peri‐operative management including anaesthesia, invasive monitoring and the multi‐modal approach to his peri‐operative management using a combination of intravenous analgesics, bilateral brachial plexus nerve catheters and a combined spinal and epidural. The patient made a good recovery and was able to return home from a rehabilitation facility 12 months after the operation, able to undertake many tasks himself with the aid of prosthetics.

## Introduction

Post‐amputation pain can be severe and difficult to manage with nociceptive and neuropathic elements in addition to central wind‐up. Poor quality postoperative pain control adversely impacts the patient's ability to engage with early rehabilitation from both physical and psychological perspectives as well as potentially impacting on the development of chronic pain. Various strategies for the management of post‐amputation pain have been described with differing levels of success in reducing chronic pain syndromes [1].

In this report, we describe the case of a patient undergoing simultaneous bilateral below knee and bilateral mid‐forearm amputation following irreversible ischaemic injury to all four limbs while being treated for severe sepsis in intensive care. This level of quadruple amputation is a rare procedure; we were unable to find any previously published literature describing anaesthetic management. The main anaesthetic challenge in this case was planning the management of the patient's postoperative pain.

## Report

A 47‐year‐old man with a history of obstructive sleep apnoea on nocturnal continuous positive airway pressure (CPAP), prediabetes, and depression treated with citalopram presented to hospital with severe sepsis of uncertain aetiology. He was admitted to intensive care with septic shock and multiorgan failure, and required tracheal intubation, ventilation and cardiovascular support. The underlying cause of the sepsis was not identified. He remained in intensive care for five weeks.

His case was complicated by irreversible ischaemic injuries to all four limbs, potentially secondary to the vasopressor treatment required for the septic shock. The ischaemic injury extended above the wrist in both arms and above the ankle in both legs. The decision from the vascular surgical team was that bilateral below knee and bilateral mid forearm amputations was the only appropriate management option.

A staged procedure was considered, but following multidisciplinary discussion, it was felt that, from a logistical and psychological point of view, it was more appropriate to proceed with all four amputations in the same procedure.

In the period immediately prior to surgery, the patient was suffering from pain related to the ischaemia and received regular paracetamol 1 g four times per day, gabapentin 100 mg three times per day, amitriptyline 50 mg once a day, oxycodone modified release 40 mg twice a day and oxycodone immediate release 10 mg up to once hourly.

All pre‐operative analgesics were continued through the peri‐operative period, and an analgesic plan was formulated following a patient‐centred, multidisciplinary consultation. Prior to induction of general anaesthesia, we sited a combined spinal and lumbar epidural, using 0.5% heavy bupivacaine 2 ml and diamorphine 1 mg. General anaesthesia was induced with fentanyl and propofol, and neuromuscular blockade provided with rocuronium. A tracheal tube was inserted and general anaesthesia was maintained with sevoflurane in oxygen‐enriched air.

After transfer to the operating table, a central venous catheter was placed in the right internal jugular vein and a left femoral arterial line was inserted. Bilateral axillary brachial plexus catheters were then inserted using ultrasound guidance (Sonosite X‐Porte, FUJIFILM Sonosite, Inc, Bothell, USA): a short‐axis view was obtained with a linear 6–15 MHz probe before advancing a 16G Tuohy needle in‐plane, superior to the artery. A bolus of 0.9% saline was used to confirm needle placement with spread around the artery and the catheters were then advanced leaving the tips posterior to the axillary arteries. A nerve catheter kit including a ‘Y’ connector was used to minimise the number of pumps required (Rectus Sheath kit, Pajunk GmbH, Geisingen, Germany). The block was established with 20 ml of 0.25% bupivacaine injected into the ‘Y’ connector.

Intra‐operatively, the patient received medications outlined in Table 1. Prior to emergence, the axillary catheters were topped up with 20 ml of 0.1% bupivacaine continued by infusion. Based on pre‐operative weight and the weight of the limbs, post‐operative weight was estimated at 85 kg and our calculated maximum hourly local anaesthetic dose was not exceeded. Neuromuscular blockade was assessed and reversed with neostigmine and glycopyrrolate, and the patient's trachea was extubated prior to transfer to the recovery area.

**Table 1:** Analgesic drugs administered intra‐operatively.

Drug	Bolus	Infusion
Magnesium sulphate	3.5 g	1.5 g.h^−1^
Ketamine	20 mg	10 mg.h^−1^
Clonidine	300 μg	N/A
Fentanyl	500 μg	N/A
Dexamethasone	6.6 mg	N/A
Ondansetron	4 mg	N/A

After emergence, the patient complained of pain in his legs, which settled with 100 mg tramadol and optimisation of the epidural blockade with an additional bolus of 0.1% bupivacaine 10 ml. Fentanyl was administered via patient‐controlled analgesia (PCA) device, with a bladder for activation placed in the patient's axilla (ArcoAir PCA Switch, Arcomed AG, Zurich, Switzerland); however, the patient was unable to apply enough pressure to activate it, so it was instead used as a nurse‐controlled device. The patient was transferred to the post anaesthesia care unit where he remained for 48 h prior to transfer to the ward. During this time, the magnesium infusion and nurse‐controlled analgesia were stopped and immediate release oxycodone restarted as required. The patient's pain was generally well controlled with no increase in opioid requirements compared to the pre‐operative period. The quality of the analgesia allowed engagement with physiotherapy.

The axillary catheters and ketamine infusion were discontinued on postoperative day 4. The epidural was removed on day 5. Following this, pain was controlled with oxycodone, gabapentin and paracetamol.

The patient made good progress and was transferred to a rehabilitation facility before being discharged home around 12 months after the operation. He is now able to walk short distances independently and feed and dress himself with the assistance of prosthetics. He uses touchscreen devices to interact with computer equipment. He has phantom pain from his hands and some neuropathic pain in his legs. This is well controlled with oxycodone modified release 5 mg twice a day and oxycodone immediate release 5 mg (averaging around 10–20 mg daily), pregabalin 300 mg twice daily and amitriptyline 25 mg at night.

## Discussion

There was excellent communication from the surgical team regarding this case allowing early discussion with the patient, ward and acute pain team. We had discussions regarding the surgical plan with a preference for a staged approach, which would allow more flexibility with acute pain control. This was deemed to be a worse option for several reasons including the psychological burden the staging time would place on the patient, the risk of deterioration between operations and also difficulties with timing of surgeon and theatre availability.

A pre‐operative review of the literature demonstrated very few case reports of quadruple amputation [2] and those we found reported more limited amputations. Given the extent of the surgery planned and pre‐operative pain, we felt that nerve blockade would be important in minimising postoperative discomfort and the potential for development of chronic pain as well as maximising the potential for early rehabilitation.

Optimal positioning of the perineural catheters was an important consideration. Continuous infusion of local anaesthetic via catheters has been described at each of the approaches to the brachial plexus; all with their own advantages and disadvantages [3, 4]. At the interscalene level, there is a significant risk of the phrenic nerve being blocked leading to diaphragmatic palsy, as well as Horner's syndrome without providing adequate analgesia to the ulnar nerve and parts of the median and radial nerve [5]. The supraclavicular approach can provide analgesia to the forearm but also has a risk of spread to the phrenic nerve or can be complicated by pneumothorax (less than 1:1000) [6]. The infraclavicular approach can also provide complete analgesia to the forearm, has a good volume of tissue to anchor a nerve catheter but also has a higher risk of pneumothorax when compared to axillary approach [6]. Analgesia via continuous infusion of local anaesthetic around the brachial plexus at the level of the axilla is safer with respect to pneumothorax or causing respiratory compromise via the phrenic nerve, but it is much less likely to provide analgesia to the lateral forearm via lateral antebrachial cutaneous nerve which comes from the musculocutaneous nerve [3]. We opted for axillary placement to avoid the risk of postoperative respiratory failure associated with the potential for bilateral pneumothoraces or phrenic nerve blockade associated with any other catheter placement.

The rest of the analgesic plan was informed by current practice in our institution and a review of the literature. Given the complexity of the planned surgery, we were concerned about postoperative pain resulting in both immediate and longer term complications. The patient had already been started on amitriptyline and gabapentin by the acute pain team. We gave intra‐operative clonidine which has an opioid‐sparing effect and reduces the incidence of postoperative nausea and vomiting. Ketamine has a wide range of actions and is useful both in acute pain and for reducing central sensitisation, which has a role in the development of chronic pain after amputation [7]. The addition of a low‐dose ketamine infusion to a patient‐controlled (or as in this case, nurse‐controlled) opioid infusion can improve pain control while reducing side effects [8]. Magnesium acts as an N‐methyl‐d‐aspartate receptor antagonist at a different site to ketamine and also blocks calcium channels. It can improve pain scores when given as part of multimodal analgesia [9, 10].

We used all of these adjuvant analgesics in order minimise the chances of excess sedation associated with opioids while providing the best postoperative analgesia possible and minimise the risk of developing chronic pain. In this case, either poor immediate pain control (and the resultant failure to engage with early rehabilitation) or the development of chronic pain would have been associated with worse functional and psychosocial outcomes.

We had to make various adjustments to our standard practice due to the nature of the surgery. Intra‐operatively the surgical team requested bilateral upper limb tourniquets, which restricted our options for vascular access and monitoring. Anaesthesia was induced via a cannula placed pre‐operatively in the antecubital fossa. This was removed following successful cannulation of the internal jugular vein. Our options for blood pressure monitoring were also limited; hence, a femoral arterial line was used.

Postoperatively we wanted to use a PCA device. Our usual practice involves a handset with a button. The alternative PCA switch available to us was a balloon similar to those on traditional sphygmomanometers. This turned out not to be suitable as in the immediate postoperative period when it was required, our patient was unable to apply sufficient force to activate it. The manufacturer also makes a variant of the PCA switch which is breath‐actuated, which might have been more successful.

Finally, we were running local anaesthetic infusions in both the epidural and both axillary catheters, but had also reduced our patient's body mass. We were careful to obtain a pre‐operative weight and then calculate a postoperative weight in order to set a maximum hourly bupivacaine dose, which was clearly documented in the notes to minimise the risk of local anaesthetic toxicity.
